# Unmanned Aerial Vehicle-Based Structural Health Monitoring and Computer Vision-Aided Procedure for Seismic Safety Measures of Linear Infrastructures

**DOI:** 10.3390/s24051450

**Published:** 2024-02-23

**Authors:** Luna Ngeljaratan, Elif Ecem Bas, Mohamed A. Moustafa

**Affiliations:** 1 Department of Civil and Environmental Engineering, University of Nevada, Reno, NV 89557, USA; lngeljaratan@nevada.unr.edu (L.N.); basel@nevada.unr.edu (E.E.B.); 2 Research Center for Structural Strength Technology, National Research and Innovation Agency (BRIN), Science and Technology Research Center Bd. 220, Setu, Tangerang Selatan 15314, Indonesia; 3 R&D Test Systems A/S, 8382 Hinnerup, Denmark; 4 New York University Abu Dhabi, Abu Dhabi P.O. Box 129188, United Arab Emirates

**Keywords:** UAV-based SHM, computer vision, MSER, SURF, MSAC, matching, accuracies, linear infrastructure, pipeline, seismic test, seismic performance

## Abstract

Computer vision in the structural health monitoring (SHM) field has become popular, especially for processing unmanned aerial vehicle (UAV) data, but still has limitations both in experimental testing and in practical applications. Prior works have focused on UAV challenges and opportunities for the vibration-based SHM of buildings or bridges, but practical and methodological gaps exist specifically for linear infrastructure systems such as pipelines. Since they are critical for the transportation of products and the transmission of energy, a feasibility study of UAV-based SHM for linear infrastructures is essential to ensuring their service continuity through an advanced SHM system. Thus, this study proposes a single UAV for the seismic monitoring and safety assessment of linear infrastructures along with their computer vision-aided procedures. The proposed procedures were implemented in a full-scale shake-table test of a natural gas pipeline assembly. The objectives were to explore the UAV potential for the seismic vibration monitoring of linear infrastructures with the aid of several computer vision algorithms and to investigate the impact of parameter selection for each algorithm on the matching accuracy. The procedure starts by adopting the Maximally Stable Extremal Region (MSER) method to extract covariant regions that remain similar through a certain threshold of image series. The feature of interest is then detected, extracted, and matched using the Speeded-Up Robust Features (SURF) and K-nearest Neighbor (KNN) algorithms. The Maximum Sample Consensus (MSAC) algorithm is applied for model fitting by maximizing the likelihood of the solution. The output of each algorithm is examined for correctness in matching pairs and accuracy, which is a highlight of this procedure, as no studies have ever investigated these properties. The raw data are corrected and scaled to generate displacement data. Finally, a structural safety assessment was performed using several system identification models. These procedures were first validated using an aluminum bar placed on an actuator and tested in three harmonic tests, and then an implementation case study on the pipeline shake-table tests was analyzed. The validation tests show good agreement between the UAV data and reference data. The shake-table test results also generate reasonable seismic performance and assess the pipeline seismic safety, demonstrating the feasibility of the proposed procedure and the prospect of UAV-based SHM for linear infrastructure monitoring.

## 1. Introduction

Linear infrastructures, such as highways, roads, railways, tunnels, and pipelines, are critical for the transportation of products and the transmission of energy. Therefore, a safe design and frequent inspections are essential to ensuring service continuity throughout their entire lifespan. The possibility of being exposed to natural hazards such as earthquakes should also be considered not only in the design and construction but also in the maintenance phases. Among linear infrastructures, the condition or seismic monitoring of natural gas pipelines is slightly more critical since their network may be constructed through several terrains with different seismicity, leading to different hazard exposures [1]. During condition monitoring, their material deterioration or structural degradation due to sustained loads, such as internal pressure, or as a result of thermal expansion can be identified early and resolved rapidly. However, occasional loads like earthquakes may lead to a sudden and significant loss of energy supply and even fire hazards, as previously experienced in the 1994 Northridge earthquake [2]. The investigation revealed that the seismic hazards contributing to the pipeline failure were mainly caused by permanent ground deformation and seismic wave propagations [3]. The traveled waveform initiated pipeline compression failure, followed by a tensile fracture that finally led to buckling. The weakest links, such as welds or other bolted connections, experienced tension failure since their design was linear with little redundancy. This indicates that pipeline networks have a lower strength level as compared to other linear infrastructure systems. Therefore, they have a high dependency on robust monitoring and a health assessment system to avoid any service interruptions and to protect them from any foreseeable risks and hazards.

The pipeline monitoring task is typically supervised by conventional controls and data acquisition systems that evaluate the pipeline condition 24 h per day, sometimes with a few seconds scanning gap, depending on the communication technology used in the field [4]. Recently, more robust monitoring techniques, such as acoustic emissions, guided waves, or wireless sensors, have also been implemented to estimate the residual life by detecting crack propagation, screening for corrosion, or identifying pressure changes that may indicate damage to the pipeline system [5,6]. The advances in the field of pipeline monitoring are shown in robotics as more flexible platforms to complete multiple works and to conduct challenging tasks in each mission. Climbing robots show efficiency in assisting pipeline inspections on land without posing a danger to human life and are also less time-consuming [7]. For a more complex environment like undersea pipes, an Autonomous Underwater Vehicle equipped with a multibeam echosounder and forward-looking sonar provides high efficiency in the inspection of subsea pipelines [8]. 

An intelligent robotic system with functions that are not only useful for condition monitoring but also beneficial for pipeline seismic hazard mitigation due to its airborne operation is provided by an unmanned aerial vehicle (UAV), publicly known as a drone. It is capable of capturing high-quality geospatial data independently of the ground movement, perfectly isolating them from other disturbances in the mission environment. Therefore, for monitoring pipelines before, after, or during seismic events, the UAV visibly outperforms its other robotic instrument counterparts. Prior UAV studies have shown that they can be operated for pipeline condition monitoring, for example, to capture gas leakage and emissions [9,10,11,12]. The vast development of computer vision algorithms also supports either laboratory or field experiments on UAV-based SHM. Generally, they are used to detect special features of the tested object through pattern recognition and matching algorithms. In the Digital Image Correlation (DIC) method [13], for example, an algorithm that uses a correlation function to allow the measurement of the surface displacement, deformation vector fields, or strain maps was used in the work by [14]. A drone-based stereo DIC was developed and tested on a prestressed concrete tie for high-speed rail application as a proof of concept for the successful integration of DIC and drone technologies. Automatic feature detection algorithms such as the Scale-Invariant Feature Transform (SIFT) [15] or Speeded-Up Robust Features (SURF) [16] enable the extraction of feature points based on a 2D discrete wavelet transform for SIFT and a Hessian matrix for SURF [17], which has also been used to analyze UAV videos. Matching algorithms, such as the greedy nearest neighbor, optimal fair or full, or exact algorithm, are selected to match features between images depending on the matching goal. False matching may occur, which should be filtered using an optimization algorithm such as random sample consensus (RANSAC) or its variants, namely, the M-estimator SAC (MSAC) [18], Progressive SAC (PROSAC) [19], or Maximum Likelihood Estimator SAC (MLESAC) [20] algorithm, of which selection is based on either its accuracy, speed, robustness, or optimality [21]. 

There are still research gaps to be explored and essential topics to be covered to support UAV-based SHM, especially for linear infrastructures. First, there are practical gaps related to the UAV potential for seismic monitoring aiming at the safety assessment of linear infrastructures. Prior works have focused only on UAVs for the inspection of tunnels [22], railways [23], power lines [24], or roads [25]. For pipeline monitoring, previous studies have only explored post-seismic actions, for example, UAV mapping that shows the surface ruptures causing pipeline leakage [26] or a UAV for pipeline post-recovery reconnaissance [12]. Second, methodological gaps in a simple yet practical method to assess the measurement accuracy of UAV data have been identified. Some challenges have been addressed, primarily on UAV stabilization, positioning accuracy, and measurement accuracy [27,28,29]. They become critical issues for intelligent UAV-based vibration monitoring purposes [30,31], as they affect data accuracy due to image distortions and misalignments. However, only UAV control, like estimating motion based on an Inertial Measurement Unit (IMU) [32] or an image-processing technique using a calibration method [33], has been adapted as a solution. 

Prior works have provided valuable insights into the potential benefits of deploying UAV-based SHM; however, further study is necessary to address previous gaps and improve our understanding in this field. Therefore, the main contributions of this study are the exploration of the UAV potential for the seismic vibration monitoring of linear infrastructures, focusing on pipeline systems, with the aid of several computer vision algorithms and the investigation of the impact of selecting several parameters for the applied computer vision algorithms on the feature matching accuracy. The goal is to deploy UAV-based SHM for seismic and safety assessments of linear infrastructures. The remainder of this paper is organized as follows. In Section 2, computer vision procedures and their relevant parameters are presented and their accuracies are verified. The small-scale validation experiment is given in Section 3. The pipeline application on a shake table is given in Section 4. Conclusions are drawn in Section 5. 

## 2. Computer Vision Procedures for UAV-Based Seismic Structural Health Monitoring 

**Figure 1:**
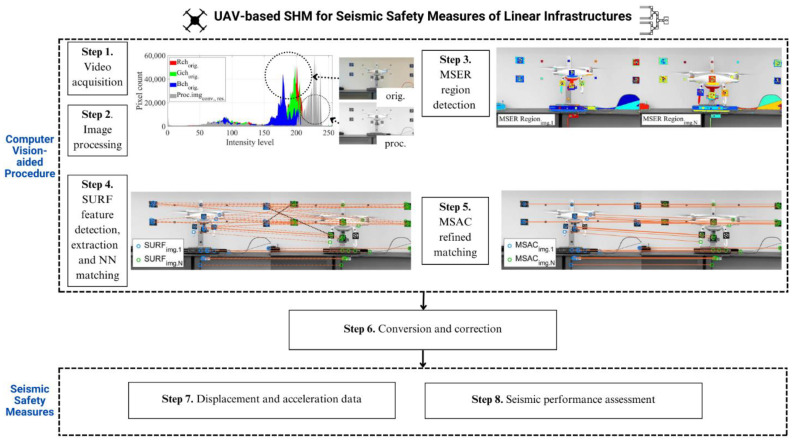
Proposed computer vision procedures for UAV-based seismic SHM for linear infrastructures.

A systematic approach using computer vision algorithms for the seismic SHM of linear infrastructures is proposed in Figure 1. A camera equipped with a UAV faces the measurement points on the object, focusing on the distributed artificial targets that are distinctive from their surrounding environment. These optical targets are based on the design of Schneider [34], in which the target centers are typically found using the ellipse-finding algorithm. They are detected, recognized, and matched later using different algorithms, as proposed in Figure 1. Depending on the field of view, the UAV should fly in front of the object, like in the example in Figure 1, or above the object, as shown later in the validation test and pipeline shake-table tests. Similar to vision-based vibration SHM using steady cameras, as previously studied by the authors [35,36,37,38,39], the UAV camera should also be kept stable, and the UAV body should not drift while monitoring the tests; otherwise, they will affect the data accuracy. Therefore, this study proposes conversion and correction steps between two key steps for UAV-based seismic SHM, i.e., the computer vision-aided procedure and seismic safety measures, as shown in Figure 1, with the details given in the next subsections. 

### 2.1. Video Acquisition and Image Processing

A UAV and its components, including the gimbal and the camera, should be calibrated before each mission following the system requirements. In each mission, the UAV records a single or multiple videos based on the number of tests or its battery life. The video is then extracted into continuous images, with the total number of images depending on the camera sampling rate and the testing duration. For a UAV camera that is set to record colored videos, the colored images are processed into grayscale to comply with the feature extraction algorithm’s requirements, as detailed in the next subsection. An illustration of the effect is shown as a histogram in Figure 1. When the RGB channels from the original image are transformed into grayscale, the pixel distribution is more stretched over the gray-level intensity. The pixel distribution shifts more to brighter areas, while in darker areas, the pixel counts are slightly reduced. The quality of underexposed or low-contrast images is then improved using the Contrast-limited Adaptive Histogram Equalization (CLAHE) method [40], which was studied previously by the authors [39], along with several image enhancement algorithms to ensure that their impacts are insignificant to the data accuracies. Next, each image is processed continuously using region detection as well as feature detection, extraction, and matching algorithms.

### 2.2. MSER Detector

The basis of fundamental computer vision tasks such as 3-D reconstruction, stereo matching, or object tracking relies on the selection and detection of regions of interest and local features, followed by finding their correspondence in the next image. Artificial targets attached to the structure are already distinguishable and easy to track within the image sequences; thus, they serve as elements and are further called distinguished regions (DRs) in this paper. They are defined by an extremal property of the intensity function in the DR, and their outer boundaries are identified as Maximally Stable Extremal Regions (MSERs), with more details in Matas et al. [41]. As compared to other region detectors, MSER has major advantages, as it is applicable for hardware operations and has high repeatability with correct identification [42,43,44]. Five parameters of MSERs should be determined, i.e., maximum and minimum areas with their variations, delta or threshold delta, and minimum diversity. Maximum and minimum areas are regions that can be considered stable with an increasing threshold delta. The minimum diversity is the value between two regions that discards the lesser diversity value. The threshold delta is the variation between gray levels for each region detected by the MSER. Each component of the MSER area increases monotonically with each increasing threshold delta. The variation in the threshold delta must be less than the maximum variation and must be a local minimum, and the diversity of overlapping MSERs must be greater than the minimum diversity to be accepted as an MSER. Furthermore, these parameters are also subjected to a constraint that they must be a positive number.

A brief explanation of the MSER algorithm is provided as follows. As mentioned previously, each RGB image is first converted to grayscale, so the intensity range is now within the range of 0 (black) to 255 (white) for 8-bit images. Then, it is segmented using intensity thresholding with certain values such that if the gray level of the image pixel is smaller than the threshold, then it is set to zero, and otherwise, it is set to one. The DR with the highest gray level is called the extremal region, Q, which is maximally stable if the variation rate of the extremal region $q\left(i_{j}\right)$ has a local minimum following the discrete approximation in Equation (1). The threshold increment ∆i  is defined by the difference $i_{j}-i_{j}-1$. These stable regions are selected as the output by selecting those with the highest stability, with an example shown in Figure 1.

(1) $$q\left(i_{j}\right)=\frac{\left\|Q\left(i_{j}\right)-Q\left(i_{j}-1\right)\right\|}{\left\|Q\left(i_{j}\right)\right\|}$$

### 2.3. SURF Descriptor 

The next challenge after detecting DRs is to keep the most visible features in those regions, such as edges, corners, blobs, etc. A local descriptor is built from these features that should stay constant under numerous disturbances, like geometric transformation, noise, or photogrammetric changes. The SURF algorithm was selected as the feature extraction descriptor in this study due to its capability to reduce computational complexity [45], which mainly consists of four steps. First, the integral image that represents the input image is created. A Hessian matrix is built, and its determinant is used to detect blob-like features, followed by generating descriptors. The properties of SURF are shown in Equation (2), which symbolizes them as a Laplacian in which $\left(x,y\right)$ is the location of point X in image I. Based on Equation (2), the SURF detector keeps the blob-like features in the image and differentiates between bright blobs detected on a dark background and vice versa. The CLAHE method, as mentioned in the previous subsection, accelerates this process since the image quality improves as the image enhancement algorithm is applied. Blob features are detected by convolving the source image with the determinant of the Hessian $\left(DoH\right)$ filters, having the 2-D Gaussian second-order derivatives $G_{xx}$ and $G_{yy}$, which are then divided by the Gaussian variance, $\sigma^{2},$ as shown in Equation (3). These features are then interpolated to sub-pixel accuracy. Two descriptor vectors are extracted in this step, i.e., 64- and 128-dimensional descriptors, which are later labeled as SURF 64-D and SURF 128-D. They are based on the sums of wavelet responses. Lastly, salient features associated with each interest point are stored. A detailed analysis of the SURF algorithm can be found in [46].

(2) $$sgn\left\{G_{xx}\left(x,y,\sigma \right)+G_{yy}\left(x,y,\sigma \right)\right\}=\left\{\begin{array}{c} +1\\ -1 \end{array}\begin{array}{c} bright\;blob\;over\;dark\;background\\ dark\;blob\;over\;bright\;background \end{array}\right.$$

(3) $$DoH\left(x,y,\sigma \right)=\frac{G_{xx}\left(x,y,\sigma \right)\cdot G_{yy}\left(x,y,\sigma \right)-G_{xx}{\left(x,y,\sigma \right)}^{2}}{\sigma^{2}}$$

### 2.4. Refined Matching

After feature detection and extraction using SURF, searching for the most similar matches for local features in image data sets becomes the next computational challenge. The matching step selected in this study is performed by making exhaustive comparisons of SURF vectors using the Euclidean distance combined with the K-nearest neighbor (KNN) method. KNN matching is searched in the metric space M. As shown in Equations (4) and (5), the problem consists of pre-processing the set of points $P=\left\{p1,p2,\ldots ,p_{n}\right\}$ such that the operation NN $\left(q,P\right)$ can be executed effectively with the query point q ∈M. The KNN method was performed in this study, as it constantly returns precisely K neighbors when there are at least K points in P and also depends on the number of returned NNs with their respective distance to q. The KNN is defined as the set A following Equation (4), in which A should satisfy the condition stated in Equation (5). The accuracy of KNN matching is computed using Equation (6) and is the percentage of the number of correct matches over the number of detected features. This equation is also used to compute the accuracy of refined MSAC.

(4) $$KNN\left(q,p,K\right)=A$$

(5) $$\left|A\right|=K,A\subseteq P$$

(6) $$\mathrm{Accuracy}\;(\%)=\frac{Number\;of\;correct\;matched\;features}{Number\;or\;detected\;features}\times 100$$

Practical applications of the KNN algorithm open the possibility of returning approximate rather than exact matches, as they are imposed to enhance the computational speed. The return matches are still close to the exact neighbors, with several false matches. The existing methods of refined matching are categorized based on their statistical, function, or graph models. Among them, the RANSAC algorithm has been widely used due to its robustness [47]. Otherwise, selecting one of its variants, such as MSAC, to improve model accuracy or enhance iteration efficiency is also another option. This study selected an MSAC algorithm that follows the random sampling consensus (RANSAC). On the basis of statistics, MSAC evaluates the hypothesis based on the minimum correlation and interrelation, followed by characterizing the error distribution as a hybrid model. The fitness of the corresponding points defined by the MSAC algorithm is computed using Equation (7), in which the number of random trials N is set to 500 to find outliers. The distance of corresponding points, $d\left(x_{i},y_{i}\right)$, from the threshold t is determined from the fundamental matrix. Inliers from two images in MSAC are mapped by a geometric transformation matrix, in which the transformation type is determined based on similarity in this study. The results of geometric transformation rely on the number of matched features such that more matching features indicate the higher efficiency of the transformation.

(7) $$measure\;of\;fitness=\overset{N}{\underset{i}{\sum }}\min\left(d\left(x_{i},y_{i}\right),\;t\right)$$

### 2.5. Conversion and Correction 

After refined matching using the MSAC algorithm, the circle center of the artificial target is detected, and the step is repeated for all image sequences. For the image sequence at time step $t_{0},\;t_{1,}\ldots ,t_{n}$, the aforementioned methods are applied separately to each image. The displacement is calculated by subtracting the location of the circle center, $x_{i},\;y_{i}$, to the location at the reference time $t_{0},$ i.e., $x_{0},\;y_{0},$ which results in pixel units. The physical dynamic displacement is calculated by multiplying a scale factor S, as shown in Equation (8), by the pixel displacement. It follows the pinhole camera model, in which $d_{i}$ is the image dimension, and $d_{p}$ is the physical dimension of the targets. It assumes that as the camera’s optical axis is perpendicular to the object surface, then all features on the object plane can be equally scaled down into the image plane.

(8) $$S=\frac{d_{i}}{d_{p}}$$

The next step is correcting the displacement data for any internal and external factors that may contribute to UAV drift or movement [48]. During a mission, a single UAV without any additional payloads may experience drifting or movement. If it is significant, then it will show specific trends in the displacement data, as shown in a previous study [48]. Therefore, background subtraction is used to separate the UAV’s drift from the specimen’s movement to stabilize it and obtain clearer vibration data.

### 2.6. Seismic Safety Measures

The final step in this procedure is to measure the seismic safety of the structure using the peak displacement and frequency content of the structure generated by the acceleration response. For validation tests, the autoregressive (AR) covariance algorithm is used to obtain the PSD spectra of the specimen, as the applied loads are simply harmonic waves. Pipeline tests use white noise loading to generate table displacement and structure acceleration, which are set as the input and output, respectively, of the covariance-driven Stochastic Subspace Identification (SSI-Cov) algorithm. The SSI-Cov algorithm is a well-known representation of a state-space model that processes response or output data into a covariance function. More details of SSI-Cov can be found in [49], with applications on vision-based SHM in [37,50]. The important modal parameters, i.e., frequency and damping, are computed using this algorithm. The shifting of these values before and after earthquake tests is used to indicate whether any damages exist in the pipeline structure.

## 3. Validation Test on Aluminum Bar

### 3.1. Specimen Layout and Instrumentation

**Figure 2:**
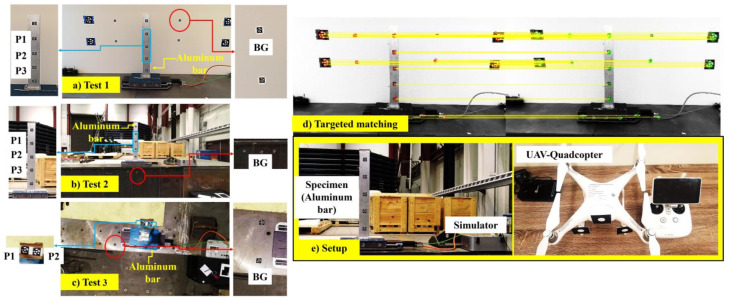
Selected features ($P_{1},P_{2},P_{3},$ and BG) from Test 1 (**a**), Test 2 (**b**), and Test 3 (**c**) with the setup on the simulator (**e**). An example of targeted feature matching with no errors (**d**) in Test 1.

The proposed framework was experimentally validated using three tests, with the layout for each test shown in Figure 2a–c. The test object was an aluminum bar with the dimensions 100 mm × 10 mm × 900 mm placed on a simulator, as shown in Figure 2e. The simulator excited the sinusoidal loading for a rigid test model. Monitoring was conducted by a quadcopter-type UAV, as shown in Figure 2e, with the specifications listed in Table 1. Figure 2d shows an example of correct pairs between images as the final result of the proposed procedure in Figure 1. It is the targeted matching showing that all features are correctly detected, tracked, and matched between images that must be achieved with all image series in each test. To present the results, the displacements of features $P_{1},P_{2},P_{3},$ and BG are selected. The background (BG) is used as a control point for correction if the UAV drifts or moves during the tests. Before the tests, the gimbal and cameras were first calibrated following the manufacturer’s recommendations. For all tests, monitoring was set at 30 frames per second (fps), i.e., a 30 Hz sampling rate, and utilized the full-resolution ROI of the UAV cameras, i.e., 3840 × 2160 pixels. The UAV was operated using the controller shown in Figure 2e such that it was navigated to be positioned in front of the specimen for Tests 1 and 2. For the third test, it flew above the specimen, recording the plan view, similar to the position while monitoring the pipeline test. 

### 3.2. MSER and Threshold Delta Variations 

**Figure 3:**
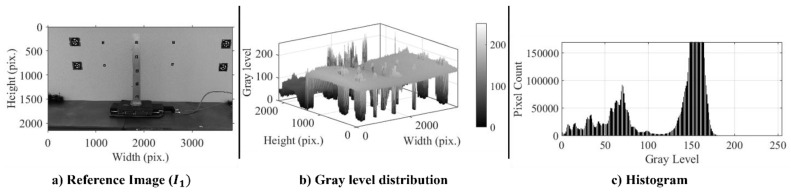
Gray-level distribution and intensity.

Following the proposed computer vision approach in Figure 1, the first procedure after converting the test video to an image series is transforming RGB channels into grayscale. The total number of images from a 36 sec test duration with a 30 fps sampling rate is 1079 images. To visualize the image characteristics, an example of the first or reference image from Test 1, $I_{1}$, with the associated gray-level distribution and histogram is given in Figure 3. The processed image has a width of 3840 pixels and a height of 2160 pixels, and the gray level is distributed among these pixels, ranging from the darkest gray value of zero to the brightest value of 255. The gray distribution of the reference image shows that most of the pixels are localized within a very low to medium gray-level intensity. The histogram shows several peaks near gray level 150 from the light background.

**Figure 4:**
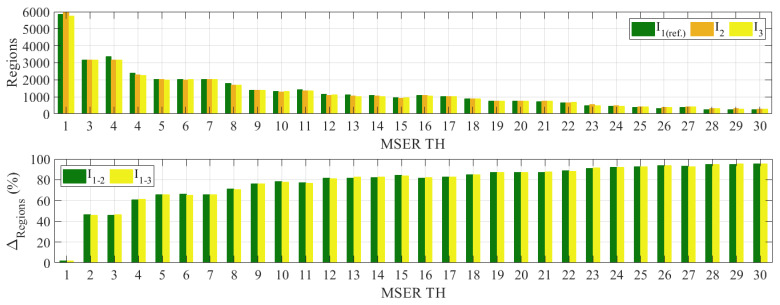
Detected regions (**top**) and differences from reference image (${∆}_{regions}$ %, **bot**.) with respect to MSER threshold delta variations (MSER TH).

**Figure 5:**
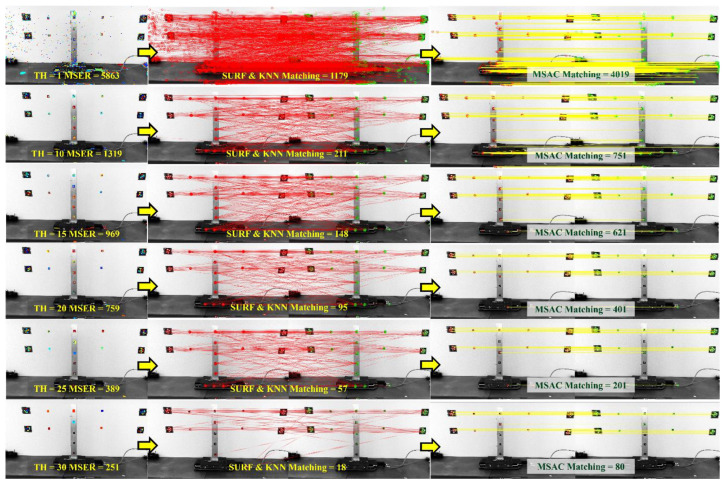
Detected MSERs and correct pairs from SURF, KNN, and refined MSAC matching concerning threshold delta variations using reference and second images from Test 1.

The MSER detector is then applied, and the five parameters explained in Section 2.2 are determined. The minimum and maximum areas of a connected component to be considered regions and, later, the expected feature size are set to 10 and 10,000 pixels, respectively. The maximum area variation between extremal regions is set to 0.25 at varying intensity thresholds. As for the MSER threshold delta (MSER TH), values ranging from 1 to 30 are selected for the thresholding process. These values are further used to explore the effect of the SURF size and threshold, as well as the MSAC threshold, to achieve accurate matching. Table 2 shows the maximum and minimum number of detected regions and extracted features of images from Test 1. Almost 6000 regions can be detected using MSER TH = 1, while a minimum of 251 regions are generated if MSER TH = 30 is selected. As the TH increases, the number of detected MSERs decreases, affecting the number of correct pairs in SURF and MSAC, as will be described in the next subsection. Figure 4 shows the number of regions detected by MSER and the difference between detected regions, ${∆}_{regions}$, of the reference image and images 2 and 3, $I_{1-2}$ and $I_{1-3}$, based on MSER TH. Figure 5 illustrates the regions detected by MSERs at TH 1, 10, 15, 20, 25, and 30. As shown in the example from Test 1 in Figure 5, 251 MSERs are detected at TH = 30; however, only 80 correct pairs are left after refined matching by MSAC. Furthermore, the algorithms detect fewer features of interest as compared to the targeted features and correct matching in Figure 2d. Therefore, the maximum MSER TH that can be applied from the three tests is TH = 30. 

In Figure 4, the example is taken from the results of Test 1, in which $I_{2}$ and $I_{3}$ are images recorded in the middle and at the end of the test. Figure 4 shows that at MSER TH = 1, $I_{1}$ detects 5863 regions, while $I_{2}$ and $I_{3}$ identify 5965 and 5746 MSERs, respectively. These numbers decrease significantly at MSER TH = 2, detecting around a 46% difference compared to TH = 1, with 3161, 3154, and 3160 regions for $I_{1}$, $I_{2}$, and $I_{3}$, respectively. As the threshold range increases, the detected regions become fewer, and only 251, 284, and 285 regions are detected for $I_{1},$ $I_{2},$ and $I_{3}$ at MSER TH = 30. These regions differ by 95% when compared to those identified at MSER TH = 1. 

### 3.3. Effect of SURF Dimension and KNN Threshold in Matching Accuracy

After detecting MSERs, the interest points in each image are extracted using SURF. It uses a box filter to estimate the Gaussian derivative, in which strong key points are detected by the determinant of the Hessian matrix. The selected SURF descriptors in this study are vectors with 64 and 128 dimensions, i.e., SURF 64-D and 128-D. The SURF vectors of interest points are calculated with a ratio threshold that is set to a maximum of one to minimize ambiguous matches. The matching process uses the Euclidean distance combined with KNN. The impact of the KNN threshold and SURF dimension selections on matching accuracy is also explored in this subsection. These algorithms are applied consecutively following the proposed procedure in Figure 1, and their results are strongly dependent on one another. Therefore, their explanation is combined in this subsection. 

**Figure 6:**
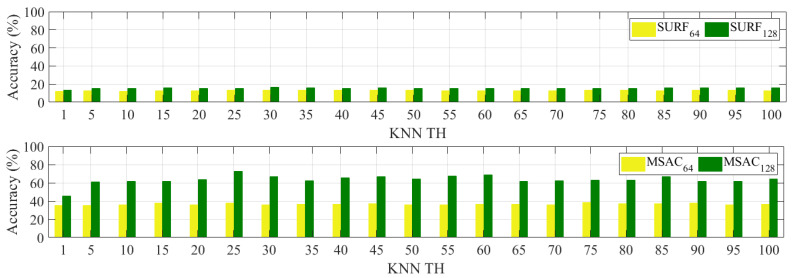
Percentage of correct matches (accuracy (%)) based on SURF 64-D and 128-D and KNN threshold variations (KNN TH).

Following the steps in Figure 5, the first observation is the effect of the SURF dimension selection and KNN threshold. An example is provided using $I_{1-2}$, and MSER TH is kept constant at 15 for this purpose. The first variation is taken for SURF 64-D and 128-D. The second is the KNN threshold variation, which is checked for thresholds of 1 to 100, with five threshold increments. Interest points are first extracted from each image after MSER detection. The MSER threshold that was selected as 15 results in 969 regions, as shown in Figure 5. The SURF feature vectors of the set of interest points are then extracted and result in 969 interest points per image following MSERs. Figure 5 illustrates an example of SURF and KNN matching for SURF 128-D, in which 148 correct points are matched at TH = 15, about 15.3% of the number of detected points. The results for both dimensions with their associated matching thresholds are given in Figure 6, and their averages are presented in Table 3. The accuracy is also checked for each dimension when the matching is refined using the MSAC algorithm, and the difference between the two dimensions is presented in Table 4. 

The effect of the KNN threshold selection is assessed first using Figure 6 and Table 3. Figure 6 shows the percentage of the KNN matching accuracy from thresholds 1 to 100 with five threshold increments based on SURF and its combination with MSAC using the dimensions 64-D and 128-D. Table 3 presents the maximum, minimum, and mean percentages of Figure 6. From these results, it is clear that selecting a higher KNN threshold does not improve the matching accuracy when it is combined either with SURF only or with MSAC. For SURF 64-D, the difference between maximum and minimum values is only 1.8%, while 2.8% is computed for SURF-128D. When integrating SURF and KNN with MSAC, about a 3.2% difference in accuracy is obtained for MSAC-64D. Meanwhile, about an 11.8% difference in accuracy is calculated between the maximum and minimum values for MSAC-128D. More analysis is then necessary to further examine the impact of using SURF 128-D over 64-D.

Table 4 presents the difference between SURF 128-D and 64-D for each algorithm as ${∆}_{SURF}$ and ${∆}_{MSAC}$ with their corresponding KNN thresholds. These values are calculated from Figure 6. The impact of using a higher SURF dimension in improving the accuracy of the matching is lower when only relying on KNN. For example, when using a threshold = 1, the two dimensions only differ by about 1.9%, and the difference then slightly increases to 3.4% at TH = 100. The average is computed as 12.7% and 15.5% for 64-D and 128-D, respectively, before MSAC. It differs by only about 2.7% between the two dimensions. When SURF and KNN are combined with MSAC, this average increases to 36.5% and 64.2% for 64-D and 128-D, about a 27.7% difference. This is about ten times higher than the result before MSAC. About a 10.2% difference in accuracy at TH = 1 is computed between the two dimensions, and then it increases up to 27.6% at TH = 100, as shown in Table 4. Modifying the SURF and KNN results with MSAC appears to affect the accuracies based on this observation. More assessments are necessary to confirm these results, focusing on the effectiveness of selecting higher SURF dimensions and combining them with MSAC. 

### 3.4. Effects of MSER Threshold, SURF Dimension, and MSAC Threshold in Improving Number of Correct Pairs and Matching Accuracy

**Figure 7:**
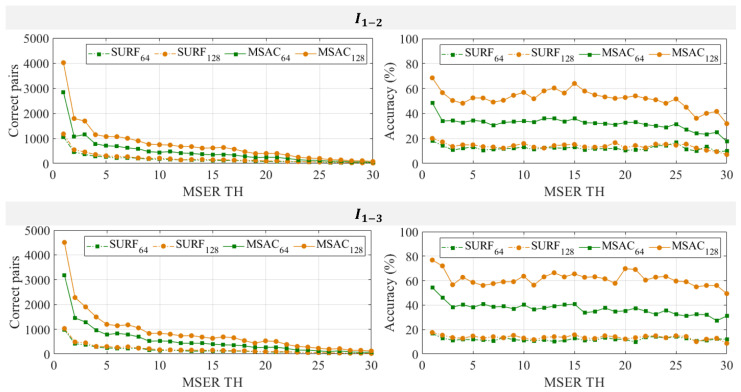
Correct pairs and matching accuracies with SURF and MSAC algorithms based on SURF 64-D and 128-D with MSER threshold delta variations (MSER TH).

The previous subsection suggests that the matching accuracy does not rely on the KNN threshold selection. A low impact is also observed when using only SURF and KNN to find perfect matches between images. This subsection focuses more on the effect of a higher SURF dimension and observes the impact of selecting MSER and MSAC thresholds. The first assessment is to check whether MSER TH selections improve the number of correct matches and accuracies if combined with SURF, KNN, and MSAC. Figure 7 shows the number of correct pair matches with their associated accuracies for different MSER TH and SURF dimensions for images 2 and 3, $I_{1-2}$ and $I_{1-3}$, respectively. Recall the results in Figure 4, where a higher MSER TH detects fewer regions, so it also affects the declining number of correct pairs, as shown in Figure 7. However, the matching accuracy only increases slightly when selecting a lower MSER TH using either only SURF and KNN or their combination with MSAC. For example, in Figure 7, for image $I_{1-2}$, when using MSER TH = 15, SURF and KNN result in 13.1% and 15.3% accuracies for 64-D and 128-D, respectively. Less improvement is observed when MSER TH = 1 is selected, as the accuracies increase by only about 5% and 4.8% for each dimension. If the combination with MSAC is considered, MSER TH = 15 results in accuracies of 35.9% and 64.1%, but they only increase slightly to 48.5% and 68.5% using MSER TH = 1 for 64-D and 128-D. 

The second assessment is to confirm the previous observation that using SURF 128-D and combining it with MSAC improves the number of correct pair matches and accuracy. For $I_{1-2}$ and $I_{1-3}$ in Figure 7, the results clearly show that using SURF 128-D with KNN and MSAC is the superior combination for increasing the number of correct pair matches as well as for enhancing the accuracy. However, SURF 64-D also performs effectively as long as the result is refined with MSAC. For example, at MSER TH = 1, 15, and 30, using image $I_{1-3}$, SURF 64-D generates 3186, 395, and 78 correct pairs, respectively, after MSAC. These numbers shift from 976, 124, and 30 correct pairs without MSAC; hence, they increase by about 69.4%, 68.6%, and 61.5%. They are associated with 54.3%, 40.8%, and 31% accuracies at MSER TH = 1, 15, and 30, rising from 16.6%, 12.8%, and 12% before MSAC, respectively. The statistics of Figure 7 are given in Table 5. It confirms that using SURF and KNN only results in 12.1% and 13.6% accuracies for SURF 64-D and 128-D, as demonstrated by the results of $I_{1-3}$. MSAC improves these results by 24.9% and 47.8%, respectively, i.e., 37% and 61.4% for SURF 64-D and 128-D after combining with MSAC. Similar performance is also given using the results of $I_{1-2}$. Combining SURF, KNN, and MSAC adds 19.6% and 37.9% accuracy to 64-D and 128-D, increasing from 12.3% and 13.9% before processing them with MSAC.

**Figure 8:**
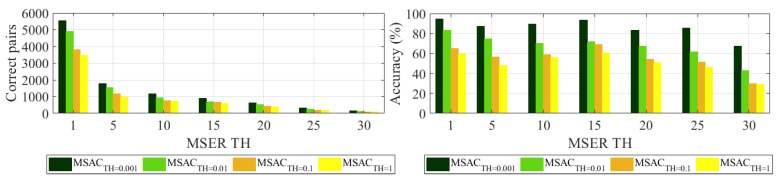
Number of correct pair matches with their respective accuracies (%) based on MSAC threshold (MSAC TH) with MSER threshold delta variations (MSER TH).

**Figure 9:**
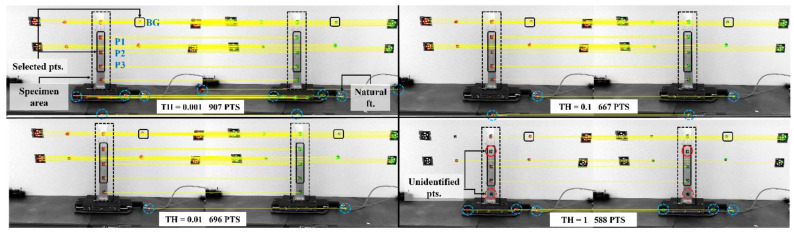
Point matching pairs with their respective MSAC thresholds. The example is taken from Test 1 and shows selected points $P_{1},\;P_{2},\;P_{3}$, and BG in the specimen area and unidentified points.

The regions in each image are first detected using MSERs. Feature extraction and matching are conducted by the proposed approaches, i.e., the SURF and KNN algorithms. The obtained results from the selected algorithms reveal that a higher SURF dimension of 128 returns more correct matches with better accuracy as compared to a dimension of 64. Moreover, when SURF and KNN are combined with MSAC, the results show a more clear improvement as compared to the results of using only SURF and KNN algorithms. Lastly, the effects of refined MSAC and the threshold are further evaluated using MSER THs of 1 to 30 with five threshold increments. The selected dimension is SURF D-128. MSAC and the threshold selections, i.e., 0.001, 0.01, 0.1, and 1, for eliminating false matches and improving accuracy are explored, as it is mentioned previously that a higher number of correct matches indicates the optimal performance of each applied algorithm. Figure 8 shows the results with the values stated in Table 6. As the MSAC threshold is selected to have lower values, the number of correct pair matches and matching accuracy are improved substantially. Selecting an MSAC threshold of 0.001 results in 5539, 1786, 1180, 907, 633, 333, and 169 correct pairs for MSER TH = 1, 5, 10, 15, 20, 25, and 30, respectively. These numbers are associated with 94.5%, 87.4%, 89.5%, 93.6%, 83.4%, 85.6%, and 67.3% accuracies, which are higher than the values when using MSAC TH = 1. Even though a lower MSAC threshold gives a more efficient performance in enhancing the number of correct matching pairs as well as a better matching accuracy, the threshold selection still needs to consider the preferred feature of interest that is located on the test object. For Test 1, the selected points to be used later in extracting the displacement are located on the aluminum bar and background, as shown in Figure 9. The example is generated using MSER TH = 15 and SURF D-128. Many extra points are refined using a lower MSAC TH, and for higher thresholds, the algorithm returns fewer points. Several natural features (marked as natural ft. in Figure 9), i.e., targetless points, are also detected in each threshold. Each threshold shows that all features of interest located in the specimen area are successfully detected, extracted, and matched, except for MSAC TH = 1. One selected point, referred to as $P_{1}$ in Figure 2, and another point located in the specimen area are not identified using MSAC TH = 1. Therefore, the suitable threshold based on the selected points is 0.001, 0.01, or 0.1. Since a threshold of 0.1 results in fewer points, which means less computational effort, yet still identifies all points of interest successfully, it can be selected as the MSAC threshold for Test 1. 

### 3.5. Test Results

**Figure 10:**
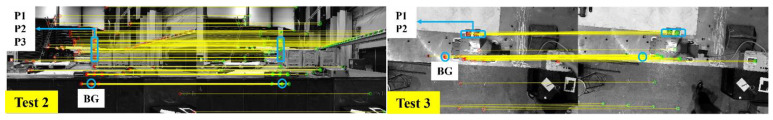
Point matching pairs from Tests 2 and 3 and selected points to measure displacement.

The previous subsection provides the implementation of the proposed computer vision procedures and explores the impact of selecting the threshold and SURF dimension. The computer vision results give an understanding of which dimension or threshold should be selected not only to accelerate UAV data processing but also to generate matched points with high accuracy. Three sin-wave tests were conducted using the aluminum bar with the testing setup shown in Figure 2 and test data given in Table 7. These tests were used to further validate the procedure before being applied to the pipeline shake-table test. Test 1 is used as an example, with comprehensive results detailed in the previous subsection. Tests 2 and 3 used a similar sampling rate of 30 fps with a shorter duration, i.e., 33 sec and 23 sec, respectively. The total number of images in each test is given in Table 7, as well as the algorithm threshold and dimension. Since the distance between the specimen and UAV is larger for Tests 2 and 3 as compared to Test 1, a smaller SURF dimension was selected to extract the preferred features, i.e., $P_{1},\;P_{2},\;P_{3}$, and BG. Surf 64-D was selected for both tests with a KNN threshold of 50, similar to Test 1. As for the MSAC threshold, Test 2 used MSAC TH = 0.1, while a threshold equal to 1 is more fitting to match the points in Test 3. An example of point matching pairs after MSAC for Tests 2 and 3 is given in Figure 10. For Test 2, selecting SURF 64-D causes many natural features located in the test environment to be detected, extracted, and matched; however, if using SURF 128-D, several points in the specimen and background are unidentified. As for Test 3, SURF 64-D is also suitable for extracting those points of interest with fewer detected natural features from the testing environment. 

**Figure 11:**
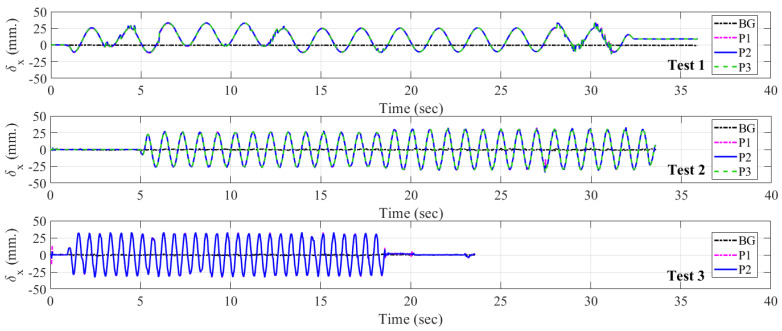
Displacement response results, $\delta_{x}$, from validation Tests 1, 2, and 3.

The displacement responses in Tests 1, 2, and 3 are given in Figure 11. The record from background points, BG, is shown in the figure, and it is subtracted from the raw displacement data of each point. The displacement of each point, $P_{1},\;P_{2},$ and $P_{3}$, is plotted together with BG data, as shown in Figure 11. The response from the three validation tests generates sinusoidal waveforms, as shown in Figure 11. The direction of the applied load to the UAV camera orientation is the in-plane direction, defined in this work as the 𝑥-direction. The peak displacements in the positive direction, $\delta_{x,max^{+}}$, and in the negative direction, $\delta_{x,max^{-}}$, of each point from each test are given in Table 8, together with their mean values. 

**Figure 12:**
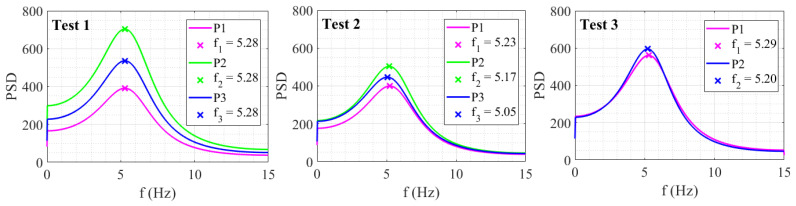
AR spectrum and natural frequency of specimen measured by validation Tests 1–3.

The verification of the test results was conducted in the frequency domain, in which the natural frequency of the specimen was measured using the acceleration response. The natural frequency of the specimen has already been measured previously by several tests [48], and the reference value taken in this study is the average value, computed as 5.31 Hz. Also, as the applied load is in the form of sinusoidal waves with a low amplitude, the specimen is expected to remain within its elastic range, causing no damage to the specimen. The frequency of the specimen in each test was generated using the autoregressive (AR) covariance algorithm and was measured from the targets $P_{1},\;P_{2},$ and $P_{3}$. The PSD spectra are shown in Figure 12, and the difference between the measured frequency and the reference frequency is given in Table 9. For Test 1, similar peaks at 5.28 Hz are computed from each point. Test 2 and Test 3 show variations with peaks at 5.23 Hz, 5.17 Hz, and 5.05 Hz calculated for $P_{1},\;P_{2},$ and $P_{3}$, while 5.29 Hz and 5.20 Hz are shown as peaks in Test 3 spectra. The mean values computed from Tests 1, 2, and 3 are 5.28 Hz, 5.15 Hz, and 5.25 Hz, respectively. The results are comparable to the reference values, which differ by about 0.53%, 2.98%, and 1.10% with respect to the natural frequency of the specimen measured from the reference data.

## 4. Pipeline Shake-Table Test

### 4.1. Testing Setup

**Figure 13:**
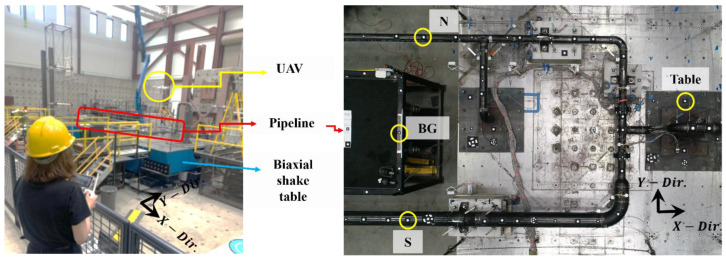
Seismic testing setup showing the UAV position during tests, pipeline position on the biaxial shake table, and selected points to generate the seismic response.

The previous section has detailed the implementation of each proposed algorithm as well as its accuracy, which was validated on a small-scale aluminum bar using three harmonic tests. The procedure was then verified on a laboratory-scale pipeline that was tested using several earthquake motions and white noise tests. The testing setup in Figure 13 shows the pipeline assembled on two shake-table tests to accommodate its length and to allow biaxial movements from shake-table excitations. The longitudinal response is defined in the x-direction, while the y-direction generates the lateral response. The UAV is operated above the shake table with the camera oriented toward the specimen, as shown in Figure 13. Due to space limitations in the laboratory, only half of the specimen with the distributed targets could be captured by the UAV. The selected targets for seismic data processing were located North (N) and South (S) with the background (BG) to consider the UAV drift during operation, similar to validation tests. The pipeline frequency response was computed using the SSI-Cov algorithm, which requires input motion obtained from the shake table; therefore, a single target attached to the shake table shown in Figure 13 was selected to generate the table displacement. A total of eight tests were conducted, with the details shown in Table 10. Four earthquake tests in the lateral and biaxial directions with increasing amplitude, together with two white noise motions before and after earthquake tests, were recorded by the UAV. The initial white noise was recorded at 30 fps, while the last ones used 60 fps to accommodate the possibility of frequency shifts due to the softening of the pipeline. 

### 4.2. Computer Vision Procedure Results

**Figure 14:**
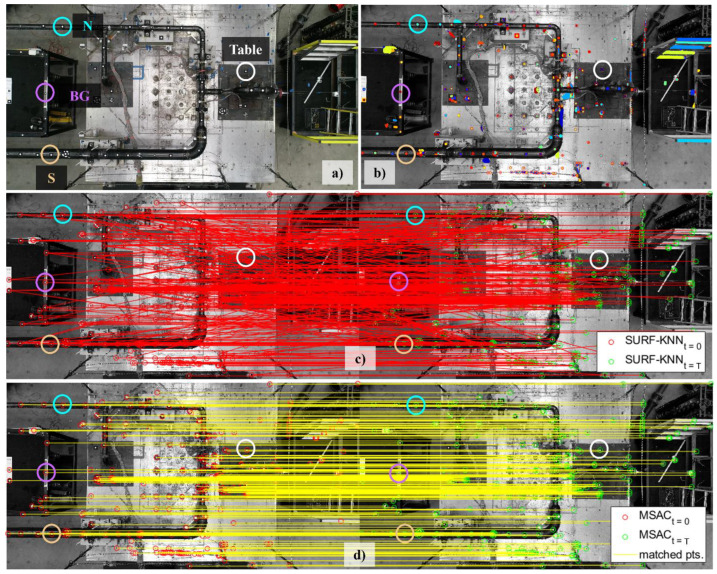
Computer vision algorithm results from pipeline test. (**a**) Feature of interests, (**b**) Detected MSER, (**c**) SURF and KNN matching, (**d**) Refined matching results using MSAC.

The UAV was operated with a mission to complete each test following the protocols in Table 10. The videos were processed to generate image sequences, and computer vision algorithms in the proposed procedures were used to detect, extract, and match the features of interest. Validation tests accelerated the process of selecting the suitable threshold and dimensions for pipeline tests, with the selected thresholds and dimensions shown in Table 10. An example of the results at the start (t=0) and at the end (t=T) is given in Figure 14 for Earthquake#1 (EQ 1). The selected MSER TH is 10, which results in 1574 regions. Using SURF 128-D and KNN TH = 50, the number of correct matches is 364 points, about a 23.12% accuracy. After refined MSAC with a threshold = 0.1, the accuracy improves to 43% as more matches are obtained, i.e., 677 correct points. These selections were used for all seismic tests. As for white noise tests, a larger SURF-128D was selected to accelerate the algorithms since more images are processed due to longer recording duration and higher frame rates. 

### 4.3. Displacement Response

**Figure 15:**
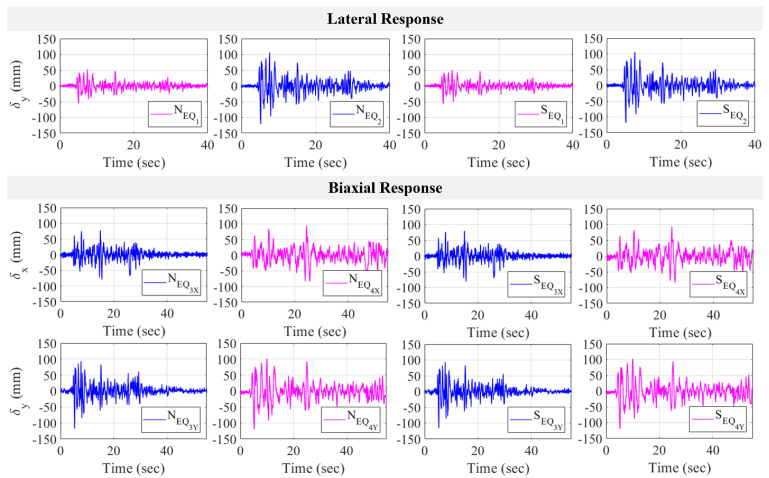
Pipeline seismic responses in lateral and biaxial directions.

After generating correct point matches, displacement data were generated from the selected points in each test. The seismic responses of all earthquake tests are given in Figure 15, with peak and average values shown in Table 11. The directions of EQ 1 and EQ 2 are lateral with increasing amplitude, which is clearly shown in the displacement response. For example, using point N, the peak displacement rises from 53.07 mm and −53.58 mm in EQ 1 to 105.73 mm and −120.54 mm as the response to EQ 2. As for pipeline behavior in the North and South directions, both show identical trends in their lateral displacement histories, with closer values for their peak-to-peak displacement. From Table 11, using EQ 2, point N results in 105.73 mm and −120.54 mm, while closer peaks of 105.47 mm and −119.04 mm are shown at point S. After uniaxial tests, two biaxial seismic tests were also performed to simulate more real-life conditions, as most structures with their components are subjected to loading in more than one direction during earthquakes. The results are shown in Figure 15, with the peak displacements tabulated in Table 11. From the figure, it is confirmed that the lateral direction is the weaker axis that generates softer responses with higher amplitudes. Similar to uniaxial tests, the pipeline also shows identical behavior on the North and South sides during biaxial seismic tests. For example, under EQ 3, point N records maxima of 81.1 mm and −82.65 mm in the longitudinal direction, while point S shows peaks at 79.31 mm and −80.93 mm. The increasing intensity of earthquake motion is observed from EQ 3 to EQ 4, which is also shown in the responses. In the longitudinal direction measured from point N, peak displacement increases from 81.1 mm and −82.65 mm during EQ 3 to 93.93 mm and −83.93 mm due to EQ 4. Similarly, a rising peak is also seen in the lateral direction, as the peak increases to 101.44 mm and −119.60 mm from 89.46 mm and −118.16 mm due to EQ 3. 

### 4.4. Frequency Response and Vibration-Based Damage Identification

**Figure 16:**
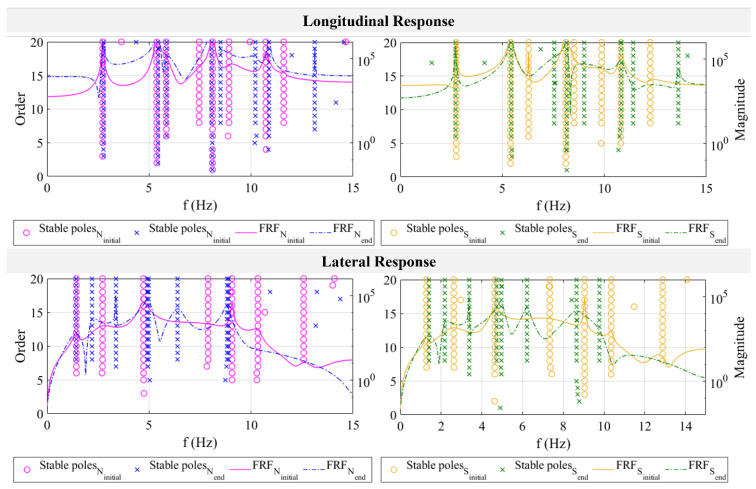
Frequency response and stabilization plots of pipeline system in lateral and longitudinal directions.

In addition to the displacement response, the frequency response is also essential to assess, as it identifies the resonant frequencies and damping of the pipeline structure. It was generated from white noise test data computed using the SSI-Cov algorithm, which uses the table motion as input and the pipeline response as output. It was measured before and after the earthquake tests to identify the initial and end states of the pipeline structure in biaxial directions and to assess whether damages occur through frequency and damping shifts. The Frequency Response Function (FRF) of the pipeline structure is plotted together with poles, as shown in Figure 16. An order of 20 is selected to generate more stable poles to assist the frequency selection. For completeness, the values of frequency and damping in Figure 16 are listed in Table 12. It is used to verify the frequency and damping results by comparing the computed values using points N and S in all five modes. It shows that the difference ranges between 0% (lateral mode-5, initial state) to 4.83% (lateral mode-3, initial state) for the frequency and slightly higher for damping, i.e., 0.25% (lateral mode-4, end state) to 5.69% (longitudinal mode-4, initial state). 

Vibration-based damage identification is conducted by analyzing whether natural frequency and damping shifts occur in both directions. Using point S, for example, the natural frequency of the pipeline in the longitudinal direction in the initial state is measured as 2.75 Hz, while after completing earthquake tests, it is calculated as 2.74 Hz. As for damping, the values do not change before and after tests at 4.41%. Small changes in frequency and damping are also monitored using the results of point N. A softer response is shown in the lateral direction, which also verifies the previous displacement results. A lower frequency of 1.43 Hz is measured using point N data with 7.61% damping. In the end state, these values remain at 1.40 Hz with 7.38% damping. In vibration-based damage detection, if the natural frequency decreases, it implies that structural damage occurs that lengthens the period of the structure. The inherent damping is also expected to be higher in the damaged state of the pipeline due to buckling or other energy-dissipating mechanisms, if they exist. Therefore, based on these evaluations, no damages occur either in the longitudinal or in the lateral direction, as frequency and damping values do not change significantly. 

## 5. Conclusions

This study aims at deploying UAV-based SHM for seismic and safety assessments of linear infrastructures by implementing computer vision algorithms to detect, extract, and match features of interest from UAV imageries. The main contributions are the filling of research gaps, i.e., the exploration of the UAV potential for the seismic vibration monitoring of linear infrastructures, focusing on pipeline systems, and the investigation of the impact of selecting several parameters for the applied computer vision algorithms on the feature matching accuracy. The study used several validation tests to quantify the effect of the algorithm threshold and dimension selection on the matching accuracy. They later accelerated data processing in pipeline seismic tests, as they provided an estimation of which values of threshold and dimension should be selected not only based on the matching accuracy but also based on the field of view. The main conclusions and key findings of this study are as follows:

- MSER TH delta and the KNN TH selection have less of an impact in improving the feature matching accuracy when they are either combined with SURF or refined further with MSAC. A lower MSER TH detects more regions, yet the matching accuracy only increases slightly, regardless of which algorithm combinations are implemented. Similarly, the selection of a lower or higher KNN threshold also has less of an impact in improving the feature matching accuracy when it is combined either with SURF or with SURF and MSAC.
- Modifying the SURF and KNN results with MSAC leads to an increased number of correct pair matches as well as improved feature matching. The selection of a higher dimension of SURF 128-D with KNN and MSAC proves to be the superior combination; however, SURF 64-D also performs effectively as long as the output is refined with MSAC. Similar to the higher SURF dimension, a lower MSAC threshold also delivers a more efficient performance in enhancing the number of correct matching pairs, as well as the matching accuracy. However, the selection needs to consider that using a lower MSAC TH may eliminate some features of interest on the test object.
- Validation tests successfully generated a harmonic response from the test object using the proposed procedure. In the frequency response, the results are comparable to the reference frequency, ranging from a 0.53% to 2.98% difference from the natural frequency measured from the reference data.
- The pipeline seismic test verifies the potential and accuracy of the proposed method for both displacement and frequency responses. The displacement responses show a similar trend in the North and South directions in uniaxial and biaxial seismic tests, with softer responses in the lateral direction. The frequency content of the pipeline computed in both directions also differs within the 0% to 4.83% range, while a slightly larger difference is measured for damping at 0.25–5.69%. By comparing the frequency and damping values in the initial and end states of the pipeline, it is confirmed that no damage occurs on the pipeline structure in either the longitudinal or lateral direction.
- Overall, it is concluded that the proposed method has potential for implementation in UAV-based vibration SHM. The combination of the MSER, SURF, KNN, and MSAC algorithms is proven to be effective and is recommended to detect, extract, and match features of interest, with their accuracies validated for the first time in this study. Their application is also verified for the first time on pipeline structures using several uniaxial and biaxial seismic tests that provide evidence of their benefits for seismic safety measures of linear infrastructures.

## Figures and Tables

**Table 1:** UAV specifications.

Aperture	f/2.8-f/11	Lens	35 mm
Battery life	30 min	Shutter speed	8-1/2000 s (mechanical) 8-1/8000 s (electric)
Control	Manual	Satellite positioning system	GPS
FOV	Forward, backward ±60° (vertical), ±27° (horizontal)	Sensor	1 inch CMOS
Gimbal stabilization	3-axis (pitch, roll, yaw)	Video transmission	720 p
Image size	3840 × 2160 pixels	Weight	1288 g
ISO	100–6400 (video)	Size (diagonal)	350 mm

**Table 2:** Maximum and minimum detected regions and extracted feature points using MSER and SURF based on MSER threshold (TH).

TH	Max.	Min.	TH	Max.	Min.	TH	Max.	Min.	TH	Max.	Min.	TH	Max.	Min.
1	5965	5746	7	2042	2015	13	1115	1023	19	757	752	25	430	389
2	3161	3154	8	1783	1691	14	1085	1029	20	759	743	26	384	335
3	3357	3151	9	1405	1391	15	969	926	21	762	735	27	419	395
4	2383	2274	10	1319	1285	16	1100	1045	22	682	637	28	309	264
5	2044	1999	11	1420	1343	17	1039	1009	23	546	490	29	303	264
6	2040	1980	12	1165	1093	18	888	878	24	481	442	30	285	251

**Table 3:** Statistics of matching accuracy of SURF and MSAC algorithms.

Algorithm and Dimension	SURF 64-D	SURF 128-D	MSAC 64-D	MSAC 128-D
Max (%)	13.6	16.4	38.5	72.8
Min (%)	11.8	13.6	35.3	61.0
Mean (%)	12.8	15.5	36.5	64.2

**Table 4:** The difference in matching accuracy between SURF 64-D and 128-D $\left({∆}_{SURF}\;\left(\%\right)\right)$ as well as between MSAC 64-D and 128-D $\left({∆}_{MSAC}\;\left(\%\right)\right)$ with their respective KNN TH (TH).

TH	${∆}_{SURF}\;\left(\%\right)$	${∆}_{MSAC}\;\left(\%\right)$	TH	${∆}_{SURF}\;\left(\%\right)$	${∆}_{MSAC}\;\left(\%\right)$	TH	${∆}_{SURF}\;\left(\%\right)$	${∆}_{MSAC}\;\left(\%\right)$
1	1.9	10.2	35	2.1	25.8	70	3.2	26.4
5	2.8	25.7	40	2.5	28.9	75	2.4	24.1
10	3.4	25.5	45	2.7	29.7	80	1.9	25.5
15	3.0	24.0	50	2.2	28.2	85	3.6	29.5
20	2.8	27.7	55	2.5	31.9	90	2.7	23.4
25	2.2	35.2	60	2.8	32.4	95	2.9	26.3
30	3.4	31.4	65	2.9	25.5	100	3.4	27.6

**Table 5:** Statistics of correct pair matching with their respective accuracies using SURF, KNN, and their combination with MSAC algorithms.

Algorithm and dimension	SURF 64-D		SURF 128-D		MSAC 64-D		MSAC 128-D	
Images	$I_{1-2}$	$I_{1-3}$	$I_{1-2}$	$I_{1-3}$	$I_{1-2}$	$I_{1-3}$	$I_{1-2}$	$I_{1-3}$
Max	1059	976	1179	1032	2847	3186	4019	4505
Min	25	29	18	22	44	72	80	124
Mean	172.6	166.8	200	190.1	468	542.1	733.3	847.2
Algorithm and dimension	SURF 64-D		SURF 128-D		MSAC 64-D		MSAC 128-D	
Images	$I_{1-2}$	$I_{1-3}$	$I_{1-2}$	$I_{1-3}$	$I_{1-2}$	$I_{1-3}$	$I_{1-2}$	$I_{1-3}$
Max (%)	18.1	16.6	20.1	17.6	48.6	54.3	68.5	76.8
Min (%)	9.5	9.8	7.2	8.8	17.5	27.3	31.9	49.4
Mean (%)	12.3	12.1	13.9	13.6	31.9	37	51.8	61.4

**Table 6:** Values of correct pair matches with their respective accuracies from Figure 8.

MSER TH	MSAC TH = 0.001		MSAC TH = 0.01		MSAC TH = 0.1		MSAC TH = 1	
	Correct Pairs	%	Correct Pairs	%	Correct Pairs	%	Correct Pairs	%
1	5539	94.5	4887	83.4	3799	64.8	3483	59.4
5	1786	87.4	1530	74.9	1153	56.4	982	48.0
10	1180	89.5	924	70.1	772	58.5	740	56.1
15	907	93.6	696	71.8	667	68.8	588	60.7
20	633	83.4	510	67.2	413	54.4	386	50.9
25	333	85.6	239	61.4	201	51.7	181	46.5
30	169	67.3	107	42.6	75	29.9	73	29.1

**Table 7:** Test data, selected threshold (TH), and dimension (D) of MSER, SURF, and MSAC algorithms.

Test #	Sampling Rate (fps)	Time (s)	Total Images	MSER TH	SURF-D	KNN TH	MSAC TH
1	30	36	1079	15	128	50	0.1
2	30	33	1009	10	128	50	0.1
3	30	23	708	10	128	50	1

**Table 8:** Peak and average displacement responses from validation Tests 1, 2, and 3.

Point	Test 1			Test 2			Test 3		
	$\delta_{x,max^{+}}$ (mm)	$\delta_{x,max^{-}}$ (mm)	$\delta_{x,\;avg}$ (mm)	$\delta_{x,max^{+}}$ (mm)	$\delta_{x,max^{-}}$ (mm)	$\delta_{x,\;avg}$ (mm)	$\delta_{x,max^{+}}$ (mm)	$\delta_{x,max^{-}}$ (mm)	$\delta_{x,\;avg}$ (mm)
$P_{1}$	33.31	−12.11	9.29	32.64	−31.23	0.11	33.04	−33.12	0.18
$P_{2}$	33.48	−13.76	9.31	33.00	−34.20	0.08	33.04	−33.08	0.18
$P_{3}$	33.15	−12.93	9.30	31.17	−32.86	0.09	-	-	-

**Table 9:** Measured natural frequency from validation Tests 1–3 and difference from reference values.

Reference Frequency (Hz)	Test 1		Test 2		Test 3	
	f (Hz)	${∆}_{f}$ (%)	f (Hz)	${∆}_{f}$ (%)	f (Hz)	${∆}_{f}$ (%)
5.31	5.28	0.53	5.15	2.98	5.25	1.10

**Table 10:** Pipeline test protocols with their selected thresholds (TH) and dimensions (D) for computer vision tasks.

No.	Test Protocols				Computer Vision Procedure			
	Test	Direction	$f_{s}$ (Hz)	Record Duration (Sec)	MSER TH	SURF-D	KNN TH	MSAC TH
1.	White Noise 1	Longitudinal —x	30	80	10	128	50	1
2.	White Noise 2	Lateral —y	30	80	10	128	50	1
3.	EQ 1	Lateral —y	60	40	10	128	50	0.1
4.	EQ 2	Lateral —y	60	40	10	128	50	0.1
5.	EQ 3	Biaxial	60	50	10	128	50	0.1
6.	EQ 4	Biaxial	60	50	10	128	50	0.1
7.	White Noise 3	Longitudinal —x	60	80	10	128	50	1
8.	White Noise 4	Lateral —y	60	80	10	128	50	1

**Table 11:** Peak displacement responses in pipeline seismic tests.

Point	EQ 1				EQ 2				
	$\delta_{y,max^{+}}$ (mm)		$\delta_{y,max^{-}}$ (mm)		$\delta_{y,max^{+}}$ (mm)		$\delta_{y,max^{-}}$ (mm)		
N	53.07		−53.58		105.73		−120.54		
S	50.39		51.64		105.47		−119.07		
Point	EQ 3				EQ 4				
	$\delta_{x,max^{+}}$ (mm)	$\delta_{x,max^{-}}$ (mm)	$\delta_{y,max^{+}}$ (mm)	$\delta_{y,max^{-}}$ (mm)	$\delta_{x,max^{+}}$ (mm)	$\delta_{x,max^{-}}$ (mm)	$\delta_{y,max^{+}}$ (mm)	$\delta_{y,max^{-}}$ (mm)	
N	81.1	−82.65	89.46	−118.16	93.93	−83.93	101.44	−119.60	
S	79.31	−80.93	93.39	−117.85	93.07	−84.19	102.06	−119.95	

**Table 12:** The dynamic characteristics of a pipeline measured from white noise tests.

**Longitudinal Mode** —x										
**Initial**										
	Mode 1		Mode 2		Mode 3		Mode 4		Mode 5	
Point	$f_{1}$ (Hz)	$∆f_{1}$ (%)	$f_{2}$ (Hz)	$∆f_{2}$ (%)	$f_{3}$ (Hz)	$∆f_{3}$ (%)	$f_{4}$ (Hz)	$∆f_{4}$ (%)	$f_{5}$ (Hz)	$∆f_{5}$ (%)
N	2.72	1.09	5.37	1.28	5.83	0.34	8.11	0.25	10.73	1.28
S	2.75		5.44		5.85		8.09		10.87	
Point	$\zeta_{1}$ (%)	$∆\xi_{1}$ (%)	$\zeta_{2}$ (%)	$∆\xi_{2}$ (%)	$\zeta_{3}$ (%)	$∆\xi_{3}$ (%)	$\zeta_{4}$ (%)	$∆\xi_{4}$ (%)	$\zeta_{5}$ (%)	$∆\xi_{5}$ (%)
N	4.63	4.75	3.96	1.76	2.22	0.45	1.49	5.69	1.36	5.15
S	4.41		3.89		2.23		1.58		1.29	
**End**										
Point	Mode 1		Mode 2		Mode 3		Mode 4		Mode 5	
	$f_{1}$ (Hz)	$∆f_{1}$ (%)	$f_{2}$ (Hz)	$∆f_{2}$ (%)	$f_{3}$ (Hz)	$∆f_{3}$ (%)	$f_{4}$ (Hz)	$∆f_{4}$ (%)	$f_{5}$ (Hz)	$∆f_{5}$ (%)
N	2.75	0.36	5.44	0.18	5.93	0.34	8.10	0.61	10.25	1.35
S	2.74		5.43		5.91		8.15		10.39	
Point	$\zeta_{1}$ (%)	$∆\xi_{1}$ (%)	$\zeta_{2}$ (%)	$∆\xi_{2}$ (%)	$\zeta_{3}$ (%)	$∆\xi_{3}$ (%)	$\zeta_{4}$ (%)	$∆\xi_{4}$ (%)	$\zeta_{5}$ (%)	$∆\xi_{5}$ (%)
N	4.66	5.36	3.60	5.51	2.64	3.65	1.56	3.70	1.42	2.74
S	4.41		3.81		2.74		1.62		1.46	
**Lateral mode** —y										
**Initial**										
Point	Mode 1		Mode 2		Mode 3		Mode 4		Mode 5	
	$f_{1}$ (Hz)	$∆f_{1}$ (%)	$f_{2}$ (Hz)	$∆f_{2}$ (%)	$f_{3}$ (Hz)	$∆f_{3}$ (%)	$f_{4}$ (Hz)	$∆f_{4}$ (%)	$f_{5}$ (Hz)	$∆f_{5}$ (%)
N	1.43	2.09	2.53	3.43	4.73	4.83	7.89	1.90	9.06	0
S	1.40		2.62		4.97		7.74		9.06	
Point	$\zeta_{1}$ (%)	$∆\xi_{1}$ (%)	$\zeta_{2}$ (%)	$∆\xi_{2}$ (%)	$\zeta_{3}$ (%)	$∆\xi_{3}$ (%)	$\zeta_{4}$ (%)	$∆\xi_{4}$ (%)	$\zeta_{5}$ (%)	$∆\xi_{5}$ (%)
N	7.61	0.39	4.06	2.17	4.15	0.48	2.45	1.61	1.45	3.97
S	7.64		4.15		4.13		2.49		1.51	
**End**										
Point	Mode 1		Mode 2		Mode 3		Mode 4		Mode 5	
	$f_{1}$ (Hz)	$∆f_{1}$ (%)	$f_{2}$ (Hz)	$∆f_{2}$ (%)	$f_{3}$ (Hz)	$∆f_{3}$ (%)	$f_{4}$ (Hz)	$∆f_{4}$ (%)	$f_{5}$ (Hz)	$∆f_{5}$ (%)
N	1.40	3.45	2.20	0.91	3.36	0.29	5.03	4.57	6.40	2.97
S	1.45		2.18		3.37		4.80		6.21	
Point	$\zeta_{1}$ (%)	$∆\xi_{1}$ (%)	$\zeta_{2}$ (%)	$∆\xi_{2}$ (%)	$\zeta_{3}$ (%)	$∆\xi_{3}$ (%)	$\zeta_{4}$ (%)	$∆\xi_{4}$ (%)	$\zeta_{5}$ (%)	$∆\xi_{5}$ (%)
N	7.38	1.73	4.51	1.33	4.51	3.63	4.02	0.25	2.55	2.35
S	7.51		4.45		4.68		4.03		2.49	

## Data Availability

The data presented in this study are available on request from the authors.

## References

[1] Wijaya H., Rajeev, Gad E. (2019). Effect of seismic and soil parameter uncertainties on seismic damage of buried segmented pipeline. Transp. Geotech..

[2] Lau D.L., Tang A., Pierre J.-R. (1995). Performance of lifelines during the 1994 Northridge earthquake. Can. J. Civ. Eng..

[3] Nair G.S., Dash S.R., Mondal G. (2018). Review of pipeline performance during earthquakes since 1906. J. Perform. Constr. Facil..

[4] Folga S.M. Natural gas pipeline technology overview, Argonne National Laboratory ANL/EVS/TM/08-5, 2007.

[5] Liu Z., Kleiner Y. (2012). State-of-the-art review of technologies for pipe structural health monitoring. IEEE Sens. J..

[6] Fan H., Tariq S., Zayed T. (2022). Acoustic leak detection approaches for water pipelines. Autom. Constr..

[7] Jiang Y., Chen D., Zhang H., Giraud F., Paik J. (2020). Multimodal pipe-climbing robot with origami clutches and soft modular legs. Bioinspiration Biomim..

[8] Zhang H., Zhang S., Wang Y., Liu Y., Yang Y., Zhou T., Bian H. (2021). Subsea pipeline leak inspection by autonomous underwater vehicle. Appl. Ocean. Res..

[9] Asadzadeh S., de Oliveira W.J., de Souza Filho C.R. (2022). UAV-based remote sensing for the petroleum industry and environmental monitoring: State-of-the-art and perspectives. J. Pet. Sci. Eng..

[10] Nooralishahi, López F., Maldague X. (2021). A Drone-Enabled Approach for Gas Leak Detection Using Optical Flow Analysis. Appl. Sci..

[11] Tian Y., Chen C., Sagoe-Crentsil K., Zhang J., Duan W. (2022). Intelligent robotic systems for structural health monitoring: Applications and future trends. Autom. Constr..

[12] Freeman M., Vernon C., Berrett B., Hastings N., Derricott J., Pace J., Horne B., Hammond J., Janson J., Chiabrando F. (2019). Sequential earthquake damage assessment incorporating optimized sUAV Remote Sensing at Pescara del Tronto. Geosciences.

[13] Sutton M.A., Orteu J.J., Schreier H. (2009). Image Correlation for Shape, Motion and Deformation Measurements: Basic Concepts, Theory and Applications.

[14] Kalaitzakis M., Vitzilaios N., Rizos D., Sutton M. (2021). Drone-based StereoDIC: Experimental validation and infrastructure application. Exp. Mech..

[15] Lowe D.G. (2004). Distinctive Image Features from Scale-Invariant Keypoints. Int. J. Comput. Vis..

[16] Bay H., Tuytelaars T., Van Gool L. (2006). SURF: Speeded Up Robust Features.

[17] Mur-Artal R., Tardós J.D. (2017). Orb-slam2: An open-source slam system for monocular, stereo, and rgb-d cameras. IEEE Trans. Robot..

[18] Torr H., Zisserman A. (1997). Robust parameterization and computation of the trifocal tensor. Image Vis. Comput..

[19] Chum O., Matas J. Matching with PROSAC-progressive sample consensus. Proceedings of the 2005 IEEE Computer Society Conference on Computer Vision and Pattern Recognition (CVPR’05).

[20] Torr H., Zisserman A. (2000). MLESAC: A new robust estimator with application to estimating image geometry. Comput. Vis. Image Underst..

[21] Martínez-Otzeta J.M., Rodríguez-Moreno I., Mendialdua I., Sierra B. (2022). Ransac for robotic applications: A survey. Sensors.

[22] Bendris B., Becerra J.C. (2022). Design and experimental evaluation of an aerial solution for visual inspection of tunnel-like infrastructures. Remote Sens..

[23] Wu Y., Qin Y., Wang Z., Jia L. (2018). A UAV-based visual inspection method for rail surface defects. Appl. Sci..

[24] Zhang Y., Yuan X., Li W., Chen S. (2017). Automatic power line inspection using UAV images. Remote Sens..

[25] Zhu C., Zhu J., Bu T., Gao X. (2022). Monitoring and Identification of Road Construction Safety Factors via UAV. Sensors.

[26] Lin Y.-S., Chuang R.Y., Yen J.-Y., Chen Y.-C., Kuo Y.-T., Wu B.-L., Huang S.-Y., Yang C.-J. (2019). Mapping surface breakages of the 2018 Hualien earthquake by using UAS photogrammetry. Terr. Atmos. Ocean. Sci..

[27] Matsuoka K., Uehan F., Kusaka H., Tomonaga H. (2021). Experimental validation of Non-Marker simple image displacement measurements for railway bridges. Appl. Sci..

[28] Wang X., Lo E., De Vivo L., Hutchinson T.C., Kuester F. (2022). Monitoring the earthquake response of full-scale structures using UAV vision-based techniques. Struct. Control. Health Monit..

[29] Han Y., Wu G., Feng D. (2022). Vision-based displacement measurement using an unmanned aerial vehicle. Struct. Control. Health Monit..

[30] Goessens S., Mueller C., Latteur P. (2018). Feasibility study for drone-based masonry construction of real-scale structures. Autom. Constr..

[31] Brinkman J.L., Davis B., Johnson C.E. (2020). Post-movement stabilization time for the downwash region of a 6-rotor UAV for remote gas monitoring. Heliyon.

[32] Ribeiro D., Santos R., Cabral R., Saramago G., Montenegro P., Carvalho H., Correia J., Calçada R. (2021). Non-contact structural displacement measurement using unmanned aerial vehicles and video-based systems. Mech. Syst. Signal Process..

[33] Wu Z., Chen G., Ding Q., Yuan B., Yang X. (2021). Three-dimensional reconstruction-based vibration measurement of bridge model using UAVs. Appl. Sci..

[34] Schneider C. (1991). 3-D Vermessung von Oberflächen und Bauteilen durch Photogrammetrie und Bildverarbeitung. Proc. Ident/Vis..

[35] Ngeljaratan L., Moustafa M.A., Pekcan G. (2021). A compressive sensing method for processing and improving vision-based target-tracking signals for structural health monitoring. Comput.-Aided Civ. Infrastruct. Eng..

[36] Ngeljaratan L., Moustafa M.A. (2020). Implementation and evaluation of vision-based sensor image compression for close-range photogrammetry and structural health monitoring. Sensors.

[37] Ngeljaratan L., Moustafa M.A. (2020). Structural health monitoring and seismic response assessment of bridge structures using target-tracking digital image correlation. Eng. Struct..

[38] Ngeljaratan L., Moustafa M.A. (2022). Uncertainty and accuracy of vision-based tracking concerning stereophotogrammetry and noise-floor tests. Metrol. Meas. Syst..

[39] Ngeljaratan L., Moustafa M.A. (2021). Underexposed Vision-Based Sensors’ Image Enhancement for Feature Identification in Close-Range Photogrammetry and Structural Health Monitoring. Appl. Sci..

[40] Lidong H., Wei Z., Jun W., Zebin S. (2015). Combination of contrast limited adaptive histogram equalisation and discrete wavelet transform for image enhancement. IET Image Process..

[41] Matas J., Chum O., Urban M., Pajdla T. (2004). Robust wide-baseline stereo from maximally stable extremal regions. Image Vis. Comput..

[42] Mikolajczyk K., Tuytelaars T., Schmid C., Zisserman A., Matas J., Schaffalitzky F., Kadir T., Van Gool L. (2005). A comparison of affine region detectors. Int. J. Comput. Vis..

[43] Salahat E., Saleh H., Sluzek A., Al-Qutayri M., Mohammad B., Ismail M. A maximally stable extremal regions system-on-chip for real-time visual surveillance. Proceedings of the IECON 2015-41st Annual Conference of the IEEE Industrial Electronics Society.

[44] Kristensen F., MacLean W.J. Real-time extraction of maximally stable extremal regions on an FPGA. Proceedings of the 2007 IEEE International Symposium on Circuits and Systems.

[45] Lee H., Jeon S., Yoon I., Paik J. (2016). Recent advances in feature detectors and descriptors: A survey. IEIE Trans. Smart Process. Comput..

[46] Oyallon E., Rabin J. (2015). An analysis of the SURF method. Image Process. Line.

[47] Wu Y., Su X., Hu X. (2016). Image stitching based on ORB feature and RANSAC. Icic Express Lett. Part B Appl..

[48] Ngeljaratan L., Moustafa M.A., Sumarno A., Prasetyo A.M., Sari D., Maidina (2023). Exploratory Study of Drone Data Stabilization with Implications in Vibration-based Structural Health Monitoring. Evergr. Jt. J. Nov. Carbon Resour. Sci. Green Asia Strategy.

[49] Peeters B., De Roeck G. (2001). Stochastic system identification for operational modal analysis: A review. J. Dyn. Sys. Meas. Control.

[50] Ngeljaratan L., Moustafa M.A. (2019). System identification of large-scale bridges using target-tracking digital image correlation. Front. Built Environ..

